# Evaluation of the *OnSite* (Pf/Pan) rapid diagnostic test for diagnosis of clinical malaria

**DOI:** 10.1186/1475-2875-11-415

**Published:** 2012-12-12

**Authors:** Abu Naser Mohon, Rubayet Elahi, Milka Patracia Podder, Khaja Mohiuddin, Mohammad Sharif Hossain, Wasif A Khan, Rashidul Haque, Mohammad Shafiul Alam

**Affiliations:** 1 International Centre for Diarrheal Disease Research Bangladesh (icddr,b), Dhaka, Bangladesh; 2 Department of Neurobiology and Developmental Sciences, University of Arkansas for Medical Sciences (UAMS), Little Rock, AR, USA; 3 Department of Biology, Memorial University of Newfoundland, St. John’s, Canada

## Abstract

**Background:**

Accurate diagnosis of malaria is an essential prerequisite for proper treatment and drug resistance monitoring. Microscopy is considered the gold standard for malaria diagnosis but has limitations. ELISA, PCR, and Real Time PCR are also used to diagnose malaria in reference laboratories, although their application at the field level is currently not feasible. Rapid diagnostic tests (RDTs) however, have been brought into field operation and widely adopted in recent days. This study evaluates *OnSite* (Pf/Pan) antigen test, a new RDT introduced by CTK Biotech Inc, USA for malaria diagnosis in a reference setting.

**Methods:**

Blood samples were collected from febrile patients referred for malaria diagnosis by clinicians. Subjects were included in this study from two different Upazila Health Complexes (UHCs) situated in two malaria endemic districts of Bangladesh. Microscopy and nested PCR were considered the gold standard in this study. *OnSite* (Pf/Pan) RDT was performed on preserved whole blood samples.

**Results:**

In total, 372 febrile subjects were included in this study. Of these subjects, 229 (61.6%) tested positive for *Plasmodium* infection detected by microscopy and nested PCR. *OnSite* (Pf/Pan) RDT was 94.2% sensitive (95% CI, 89.3-97.3) and 99.5% specific (95% CI, 97.4-00.0) for *Plasmodium falciparum* diagnosis and 97.3% sensitive (95% CI, 90.5-99.7) and 98.7% specific (95% CI, 96.6-99.6) for *Plasmodium vivax* diagnosis. Sensitivity varied with differential parasite count for both *P. falciparum* and *P. vivax*. The highest sensitivity was observed in febrile patients with parasitaemia that ranged from 501–1,000 parasites/μL regardless of the *Plasmodium* species.

**Conclusion:**

The new *OnSite* (Pf/Pan) RDT is both sensitive and specific for symptomatic malaria diagnosis in standard laboratory conditions.

## Background

While the number of malaria cases has declined in recent times, 50% of the world’s population living in 106 countries is still at risk of malaria infection. In 2010, 216 million malaria cases were recorded worldwide. Africa has the highest burden of malaria with 81% of the cases and 91% of the deaths due to malaria globally [1].

Bangladesh is a hypo-endemic area for malaria transmission with 13 endemic districts. In 2011, 52,598 malaria cases were reported in Bangladesh [2]. A large majority (95%) of malaria cases are caused by *Plasmodium falciparum* infection in Bangladesh and transmitted by a wide variety of malaria vectors [3,4].

Accurate diagnosis of malaria is the key factor not only to prevent morbidity and mortality but also to restrict the use of anti-malarial drugs to minimize the spread of drug resistance [5]. Diagnostic improvement is necessary in both economically underdeveloped malaria-endemic countries where resource is the primary barrier, and in non-endemic malaria regions, mostly the developed countries, where the emphasis to develop expertise is less of a concern due to lack of disease prevalence.

Although clinical diagnosis of malaria is paramount in resource-limited areas, at times it raises doubt due to the overlap of similar symptoms of other tropical diseases and co-infection [6]. Furthermore, there are marked variations in clinical diagnosis accuracy in different age groups, and on the seasonality and level of endemicity of malaria in different settings [7,8].

In 1904, Gustav Giemsa introduced of a novel mixture of methylene blue and eosin stain [9]. Subsequently, Giemsa-stained blood smear examination has become the gold standard for malaria diagnosis worldwide. However, malaria microscopy has two main disadvantages. Firstly, it is very difficult to implement, especially in rural areas where very basic laboratory facilities are not available. Secondly, test results vary from area to area based on individual skills, quality of equipment and the level of parasitaemia in a specimen. Even in the most favourable conditions, sensitivity and specificity are close to 80-90% [10].

Different approaches, including ELISA, PCR and Real Time PCR have been utilized to improve malaria diagnosis. Although these methods improve sensitivity and specificity and are applicable in reference settings where laboratory facilities are specialized available, these methods are difficult to implement at field level and can only be used as a standard method to validate other methods [11]. Immunochromatography-based rapid diagnostic tests (RDTs) were introduced in the early 1990s. These tests diagnose malaria using lateral flow of blood on a nitrocellulose membrane causing an immunological reaction with bound antibodies fixed on the distant location of the membrane. Currently three types of target antigens are detected in malaria RDTs by recombinant antibodies [5]. Histidine-rich protein 2 (HRP2) is the target for most RDTs that detect *P. falciparum* infection. Forms of lactate dehydrogenases (pLDH) are used to identify both *P. falciparum* and *Plasmodium vivax* infection and a variant can be used to detect all species (Pan-specific). The third target antigen is aldolase, which is Pan-specific for all malaria species. Combining any two of these three antigen detection tests into malaria RDTs allow detection of *P. falciparum* alone, *P. vivax* alone, or mixed infection [12]. Research and training in tropical diseases has introduced principles for the development and evaluation of diagnostic tests for malaria and other infectious diseases [13]. World Health Organization is also conducting evaluation programmes to improve and control the quality of RDTs [14]. Following the WHO strategy, the Government of Bangladesh has incorporated RDTs into its National Malaria Control Program (NMCP).

A number of malaria RDTs are available in local markets and are being used for diagnosis throughout Bangladesh, but their clinical proficiency needs to be evaluated. In this study, a newly available RDT, *OnSite* (Pf /Pan) (CTK Biotech Inc, USA), was evaluated compared to microscopy adjusted with nested PCR as the gold standard.

## Methods

### Study area and population

Whole blood samples were obtained from two different sub-districts of the south-eastern part of Bangladesh: Matiranga Upazila in Khagrachhari district and Ramu Upaliza in Cox’s Bazar district. Subjects were enrolled into the study at the Upazila Health Complexes (UHCs). Febrile patients identified by clinical symptoms and referred to laboratory investigations from May 2009 to December 2010 were included in this study. Samples were excluded from the study in the case of mixed infection or differential diagnosis by the standard methods (microscopy and nested PCR).

### Sample collection

Approximately 5 mL of blood was taken through venipuncture from adult subjects by an expert medical technologist. In the case of children or minor subjects, approximately 3 mL of blood was taken. Thick and thin smear slides were prepared in duplicate using two drops of blood for each sample. The remaining blood was preserved at −20°C in EDTA tube and transported to the icddr,b Dhaka laboratory in cool boxes, maintaining the temperature below 4°C using ice bags.

### Microscopy

Microscopy was conducted at the field site by experienced microscopists following the standard procedure [11] in the corresponding UHC. The microscopy results were cross checked at the icddr,b Dhaka laboratory by a second, independent, experienced microscopist.

### Rapid diagnostic tests (RDT)

*Onsite* (Pf/Pan) RDT device, produced by CTK Biotech Inc, USA, was evaluated in this study. This device has the ability to detect Pan-specific lactate dehydrogenase (pLDH) and *P. falciparum* specific HRP-2.

All RDTs were performed on six months to two years preserved samples at the Parasitology Laboratory, icddr,b Dhaka. If both LDH and HRP line or only HRP line is found then the experimental sample is considered as *P. falciparum* positive. Conversely, only LDH positive samples were diagnosed as non- falciparum, exclusively *P. vivax* in this study. These RDTs were stored between 20-25°C at the icddr,b Dhaka laboratory.

### DNA extraction

DNA extraction was done using QiaAmp blood mini kit from Qiagen, Germany according to the manufacturer’s instructions from preserved whole blood.

### Nested PCR

A modified version of the nested PCR approach described by Snounou *et al. *[15] was used to confirm the results obtained by microscopy. Taq polymerase, PCR buffer, dNTPs and magnesiun chloride were obtained from New England BioLabs Inc, USA. All reactions were carried out in Biorad C1000 thermal cycler and Nested 2 amplicons were visualized under UV light by Biorad gel documentation system in a 1.5% agarose gel after ethidium bromide staining.

### Data analysis

All data analysis was conducted in Stata version 11.0 (StataCorp, College Station Texas, USA). Sensitivity, specificity, predictive values and accuracy were calculated with their corresponding 95% confidence intervals (95% CI) by McNemar test and exact McNemar test using the ‘diagt’ command.

### Ethical approval

The study was approved by the Research Review Committee and Ethical Review Committee of icddr,b. Informed consent was obtained from all adult subjects, and assent was obtained from the legal guardians in the case of minor subjects before the collection of blood sample. Complete anonymity was maintained in all stages of the study. Good clinical and laboratory practices were followed in all the procedures.

## Results

### Enrolment

After adjusting microscopy with nested PCR, blood samples from 372 febrile patients were included in this evaluation. Of these subjects, 52.8% were male and 47.2% were female and the age of the subjects ranged from 1.5 years to 82 years with a median of 19.4 years.

### Microscopy confirmed by nested PCR

Malaria cases were confirmed by both microscopy and nested PCR. There were 229 positive malaria cases (61.6%). Among these cases, 68.1% (156/229) were infected with *P. falciparum* and 31.9% (73/229) by *P. vivax* (Table 1). The parasitaemia of *P. falciparum* ranged from 16 to 261,480 parasites/μL (IQR: 7,680-48,730) with median value of 19,960 parasites/μL. Only 3.8% (6/156) of samples had less than 100 parasites/μL while 89.1% (139/156) of samples contained 1,000 parasites/μL or more. For *P. vivax* infection, the minimum count was identical to *P. falciparum* (16 parasites/μL) whereas the maximum parasite density observed was 25,120 parasites/μL (IQR: 680–7,220) with a median parasitaemia of 1,950 parasites/μL. The majority of *P. vivax* samples had a parasitaemia greater than 1,000 parasites/μL (63%, 46/73).

**Table 1:** Summary of the results from different malaria diagnostic tests

**Test**	**Negative**	**Pf**	**Pv**	**Total positive**
Microscopy and nested PCR	143 (38.4)	156 (41.9)	73 (19.6)	229 (61.6)
*OnSite* (Pf/Pan)	149 (40.1)	148 (39.8)	75 (20.2)	223 (59.9)

### *OnSite* (Pf/Pan) rapid diagnostic test

*Onsite* (Pf/Pan) RDT was 94.2% sensitive (95% CI, 89.3-97.3) and 99.5% specific (95% CI, 97.4-100.0) for *P. falciparum* diagnosis. The sensitivity and specificity for *P. vivax* diagnosis were 97.3% (95% CI, 90.5-99.7) and 98.7% (95% CI, 96.6-99.6), respectively. Sensitivity, specificity, positive predictive value (PPV) and negative predictive values (NPV) are shown in Table 2. In terms of parasitaemia, the lowest sensitivity was observed in samples with less than 100 parasites/μL for both *P. falciparum* (50%) and *P. vivax* (80%). The maximum sensitivity (100%) was observed in samples where the parasitaemia was between 501–1,000 parasites/μL for both species. It was evident that sensitivity increased with increasing parasitaemia (Table 3).

**Table 2:** **Sensitivity, specificity, positive predictive value (PPV), and negative predictive value (NPV) of *OnSite *(Pf/Pan) in comparison with microscopy adjusted by nested PCR as gold standard**

**Method**	**Test type**	**Sensitivity (95% CI)**	**Specificity(95% CI)**	**PPV(95% CI)**	**NPV(95% CI)**	**Agreement**
*OnSite* (Pf/Pan)	Pf	94.2 (89.3 -97.3)	99.5 (97.4 - 100.0)	99.3 (96.3 - 100.0)	96.0 (92.5 - 98.1)	0.973
*OnSite* (Pf/Pan)	Pv	97.3 (90.5 - 99.7)	98.7 (96.6 -99.6)	94.7 (86.9 - 98.5)	99.3 (97.6 -99.9)	0.984

**Table 3:** **Sensitivity of *OnSite *(Pf/Pan) RDT by parasitaemia**

**Parasite count/μL**	**Number of Pf positive sample (n)**	**Number of Pv positive sample (n)**	**Pf sensitivity (95% CI)**	**Pv sensitivity (95% CI)**
1-100	6	5	50 (13.9-86.1)	80 (29.9-98.9)
101-500	5	10	60 (17.1-92.1)	90 (54.1-99.5)
501-1,000	6	12	100 (51.7-100)	100 (69.9-100)
1,001-5,000	16	25	93.8 (67.7-99.7)	100 (83.4-100)
5,000+	123	21	97.6 (92.5-99.4)	100 (80.8-100)

## Discussion

*OnSite* (Pf/Pan), a newly available RDT for malaria diagnosis, performed well in this study. *OnSite* (Pf/Pan) RDT was 96.7% sensitive and 98.6% specific with varying performance for *P. falciparum* and *P. vivax* detection. *OnSite* (Pf/Pan) RDT has inherent capability to detect all types of non-falciparum malaria but only *P. vivax* was considered in this study due to the absence of *Plasmodium malariae* and *Plasmodium ovale* samples in the study areas. In the case of *P. falciparum*, the sensitivity ranged from 50% to 100% depending on the parasitaemia.

The World Health Organization recommends at least 95% sensitivity of RDTs for low parasite counts [5]. As like many other malaria RDTs, *OnSite* (Pf/Pan) failed to achieve this level of sensitivity. However, only six *P. falciparum* and five *P. vivax* samples had parasite loads below 100 parasites/μL in this study. Therefore, the low sensitivity observed in low parasitaemia samples may have been driven by the lack of sample size and it cannot be concluded that *OnSite* (Pf/Pan) does not perform well in low parasitaemia samples.

In the case of *P. vivax,* the sensitivity consistently increased as the parasite load increased. This supports the idea of the intra-species conserved nature of pLDH [16]. For *P. falciparum,* the sensitivity did not always increase as the parasite load increased (Table 3). This occurred because one sample with parasitaemia greater than 1,000 parasites/μL and three samples with parasitaemia greater than 5,000 parasites/μL tested negative by RDT. It is possible that this was caused by deterioration of the target antigen (HRP-2) as the tests were conducted with preserved specimens. Another possibility for this observation is intra-species variation in the gene encoding the HRP gene [17] or deletion of the gene [18] among different *P. falciparum* isolates; however this was not assessed, as it was beyond the scope of this study. One negative sample was falsely tested as *P. falciparum* positive followed by another one as *P. vivax* by *OnSite* (Pf/Pan) RDT which might be due to cross reactivity of other infections, such as rheumatoid factor, heteropheline antibodies [19]. Histidine-rich protein can persist in the blood for more than one month even after a patient is cured of malaria [20], which could also be the cause for false positivity in this observation. Conversely, three confirmed *P. falciparum* samples tested positive in Pan line but HRP2 line did not appear. This might be due to inherent limitation of the device, intra-species variation in the gene encoding the HRP gene or deletion of HRP gene.

*OnSite* (Pf/Pan) RDT was also incorporated into the WHO RDT evaluation programme in Round 3 and evaluated with good score [14]. This RDT tested to have 83.8% and 85.7% detection rate/score for *P. falciparum* and *P. vivax,* respectively, when parasitaemia were 200 parasites/μL. Furthermore, it had 100% detection rate/score for species in the cases of parasitaemia at 2,000 parasites/μL or more in WHO Panel. This study also reveals an almost similar level of sensitivity especially for higher parasite load, denoting the accuracy of this RDT in identifying symptomatic malaria cases.

*OnSite* (Pf/Pan) performed better than some similar RDTs developed and widely adopted for malaria diagnosis. Parascreen Pf/Pan RDT was evaluated in different studies [20-26] where it showed maximum sensitivity of 94% and specificity of 72% for *P. falciparum* detection. For Pan detection, it showed a maximum sensitivity of 82.5% and specificity of 78.2% for symptomatic patients [26].

OptiMAL (Pf/Pan) is another similar RDT that has been implemented in both the field and the laboratory and has been evaluated several times [27-30]. Sensitivity ranged from 91.2%-95.4% for *P. falciparum* and 60.7%-100% for *P. vivax* malaria while specificity was close to 95% or above [27,28].

CareStart™ (Pf/Pan) was also evaluated in several experimental conditions [20,27,30-35]. Its overall sensitivity varies from a minimum of 88.24% [32] to maximum 95.6% [30], while the specificities were close to 95%. The highest sensitivity for *P. falciparum* diagnosis was observed at 99.4% in Sierra Leone [33] while the lowest reported was 85.6% in Ethiopia [20] with high specificity in all relevant studies. For *P. vivax* diagnosis, the highest sensitivity was 92.3% in a study in Madagascar [30] while lowest was 85% [20] in the same study in Ethiopia where *P. falciparum* detection sensitivity was lowest [29]. Specificities of *P .vivax* detection tests were always very good, above 95% in recent studies.

Compared to the performance of various other RDTs with similar characteristics, *OnSite* (Pf/Pan) can be considered an effective diagnostic tool for detecting both *P. falciparum* and *P. vivax* malaria. This test has high positive predictive value and negative predictive value which indicates that it is capable of detecting true malaria cases as well as excluding non-malaria cases with overlapping symptoms to reduce treatment burden, which happens to be the major concern since artemisinin-based combination therapy is the last oral option presently used in these areas against *P. falciparum.*

This study used frozen blood samples and well preserved devices for evaluating *OnSite* (Pf/Pan) RDT, which may not be controlled in field settings. Thus, a slight variant result may be possible in the case of fresh blood samples in ambient field settings.

## Conclusion

*OnSite* (Pf/Pan) RDT, newly manufactured by CTK Biotech Inc, USA, performed satisfactorily in standard experimental conditions. It can be utilized for diagnosis of symptomatic malaria as well as to discriminate falciparum malaria infection from vivax malaria in endemic areas and during outbreaks. However, a field evaluation is required to assess its applicability for routine diagnosis in resource-limited settings where the feasibility of microscopy is limited. Careful monitoring should be maintained at the level of manufacturing and post-marketing surveillance to assure quality in different lots.

## Competing interests

The authors declare that they have no competing interests.

## Authors’ contributions

ANM and MSA conceptualized and designed the study, collected and identified samples, analysed the data, drafted the manuscript and made final revisions. ANM, RE. MPP, KM, WAK, RH and MSA did sample analyses and made critical revision of the manuscript. MSH conducted data analysis. ANM, RE and MSA drafted the manuscript. All the authors read the final version of the manuscript and approved.
